# Silenced ZNF154 Is Associated with Longer Survival in Resectable Pancreatic Cancer

**DOI:** 10.3390/ijms20215437

**Published:** 2019-10-31

**Authors:** Felix Wiesmueller, Josephin Kopke, Daniela Aust, Janine Roy, Andreas Dahl, Christian Pilarsky, Robert Grützmann

**Affiliations:** 1 Department of Surgery, University Hospital Erlangen, Friedrich-Alexander-University of Erlangen-Nuremberg (FAU), 91054 Erlangen, Germany; Felix.Wiesmueller@uk-erlangen.de (F.W.); robert.gruetzmann@uk-erlangen.de (R.G.); 2 Department of Urology, Asklepios Hospital Weißenfels, 06667 Weißenfels, Germany; Josephin.Kopke@gmx.de; 3 Institute of Pathology, University Hospital Carl Gustav Carus, TU Dresden, 01307 Dresden, Germany; daniela.aust@uniklinikum-dresden.de; 4 Staburo GmbH, 81549 Munich, Germany; Janine.Roy@mail.de; 5 Dresden-Concept Genome Center, Center for Molecular and Cellular Bioengineering (CMCB), Technische Universität Dresden, 01307 Dresden, Germany; andreas.dahl@tu-dresden.de

**Keywords:** pancreatic cancer, PDAC, ZNF154, SLFN5, DNA methylation, methylation-specific PCR, biomarker

## Abstract

Pancreatic cancer has become the third leading cause of cancer-related death in the Western world despite advances in therapy of other cancerous lesions. Late diagnosis due to a lack of symptoms during early disease allows metastatic spread of the tumor. Most patients are considered incurable because of metastasized disease. On a cellular level, pancreatic cancer proves to be rather resistant to chemotherapy. Hence, early detection and new therapeutic targets might improve outcomes. The detection of DNA promoter hypermethylation has been described as a method to identify putative genes of interest in cancer entities. These genes might serve as either biomarkers or might lead to a better understanding of the molecular mechanisms involved. We checked tumor specimens from 80 patients who had undergone pancreatic resection for promoter hypermethylation of the zinc finger protein ZNF154. Then, we further characterized the effects of ZNF154 on cell viability and gene expression by in vitro experiments. We found a significant association between ZNF154 hypermethylation and better survival in patients with resectable pancreatic cancer. Moreover, we suspect that the cell growth suppressor SLFN5 might be linked to a silenced ZNF154 in pancreatic cancer.

## 1. Introduction

Pancreatic cancer remains one of the deadliest cancer types with an estimated median 5-year survival rate of only 3% [1]. More than 90% of pancreatic cancers are diagnosed in patients 55 years of age or older [2] and the incidence is projected to rise in the future [3]. The term pancreatic cancer usually refers to pancreatic ductal adenocarcinoma (PDAC). This histologic type represents about 90% of all pancreatic cancers and originates from the ductal epithelium of the exocrine pancreatic portion [4]. Other types of pancreatic cancer include forms that arise from the exocrine part as well, e.g., acinar cell carcinoma, or tumors that emerge from different parts, e.g., neuroendocrine tumors.

The causes of PDAC are still not completely understood. There is evidence for certain risk factors, such as tobacco smoking, chronic pancreatitis, positive family history, longstanding diabetes mellitus, or obesity [2]. Symptoms are sparsely found in patients with early disease. Unspecific findings, such as weight loss or abdominal pain, make this entity hard to diagnose [5].

The grim prognosis is due to several factors. Late diagnosis, unavailability of screening, retroperitoneal location of the pancreas, and advanced age of patients offer limited therapeutic options. On a molecular and cellular level, PDCA exhibits aggressive growth and resistance to chemotherapeutic agents [6]. Despite more than 50 years of research, advances in therapy and, ultimately, improvements of outcomes have been small. However, Yachida et al. [7] found that there is a latency period of at least 15 years for evolution from a single PDAC cell to metastatic tumor growth. Therefore, there may be ample time to diagnose and possibly cure patients with a tailored therapy if the tumor is caught at a very early stage and if its molecular characterization is understood well.

In previous studies, we proposed analysis of hypermethylation in tumor tissue as a means to spot putative genes of interest [8,9]. Recent research has disclosed DNA promoter hypermethylation of the zinc finger protein ZNF154 in a variety of tumor cell lines [10]. We hypothesized that the ZNF154 promoter is hypermethylated in vitro as well as in vivo. More specifically, we aimed to analyze pancreatic tumor tissue samples from patients who received pancreatic resection for ZNF154 hypermethylation and to further characterize the effect of ZNF154 hypermethylation in vitro.

## 2. Results

### 2.1. Promoter Hypermethylation Analysis in Different Cell Lines

**Figure 1:**
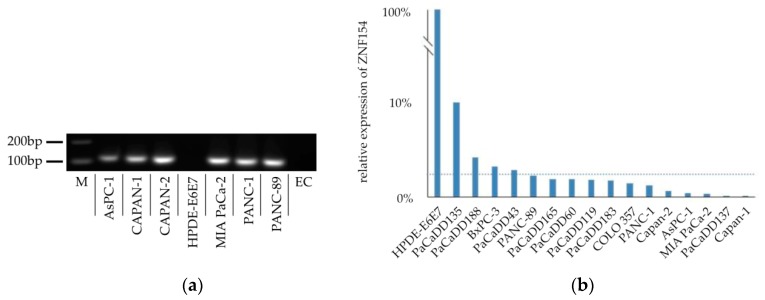
(**a**) Exemplary image of PCR gel electrophoresis checking ZNF154 hypermethylation in different cell lines (AsPC-1 cells, CAPAN-1 cells, CAPAN-2 cells, HPDE-E6E7 cells, MIA PaCa-2 cells, PANC-1 cells, TM3M4 cells; M = marker at 200 and 100 bp, EC = empty control). (**b**) Relative expression levels of RNA coding for ZNF154 found in different cell lines compared to that in HPDE-E6E7 cells (dotted line = mean relative expression of 2.3%).

We aimed to analyze the ZNF154 methylation status of different established cell lines. Purified DNA samples were extracted and subjected to sodium bisulfite conversion. PCRs using HB14 primers were performed to distinguish between true-positive sodium bisulfite-converted samples and false-positive hypermethylation status [9,10]. Samples that showed successful amplification were approved for methylation-specific PCR analysis. We found that, among other cell lines, the PANC-1 cell line was positive for hypermethylation (Figure 1a). HPDE-E6E7 cells are normal, immortalized ductal epithelial cells [11]. In our analysis, we discovered that these cells were negative for hypermethylation.

Hypermethylation of the ZNF154 promoter region had a strong inverse correlation to transcription of ZNF154 RNA [10]. We checked levels of expression of RNA coding for ZNF154 in the above-mentioned cell lines by performing real-time PCR. A ratio was calculated using the HPDE-E6E7 expression levels, which were assumed to exhibit expression levels of 100%. PANC-1 cells had a relative expression level of less than 2.3% compared to RNA levels found in HPDE-E6E7 cells (Figure 1b).

### 2.2. Patient Collective

Pancreatic specimens were obtained from patients undergoing pancreatic resection due to pancreatic ductal adenocarcinoma. In total, 86 patients were initially included. Exclusion criteria were insufficient DNA concentration of sample (< 2 ng/μL), incomplete bisulfate conversion, missing data, loss to follow-up, or postoperative survival of less than 14 days. After applying exclusion criteria, a total of 80 patients remained in the final patient collective for further examination. Table 1 demonstrates patient characteristics of this patient collective. The most common tumor size was pT3 (83.3%), with most patients having positive lymph node status (pN1 = 67.5%) while not suffering from metastasized disease (pM0 = 91.3%). Accordingly, AJCC tumor stage IIb was the most common (58.8%). Tumor differentiation found in samples was mostly classified as G2 (52.5%) or G3 (41.3%). The mean patient age was 66 years, with a mean survival of 707 days following resection.

### 2.3. Methylation Status of ZNF154 Promoter in Patient Specimens

**Figure 2:**
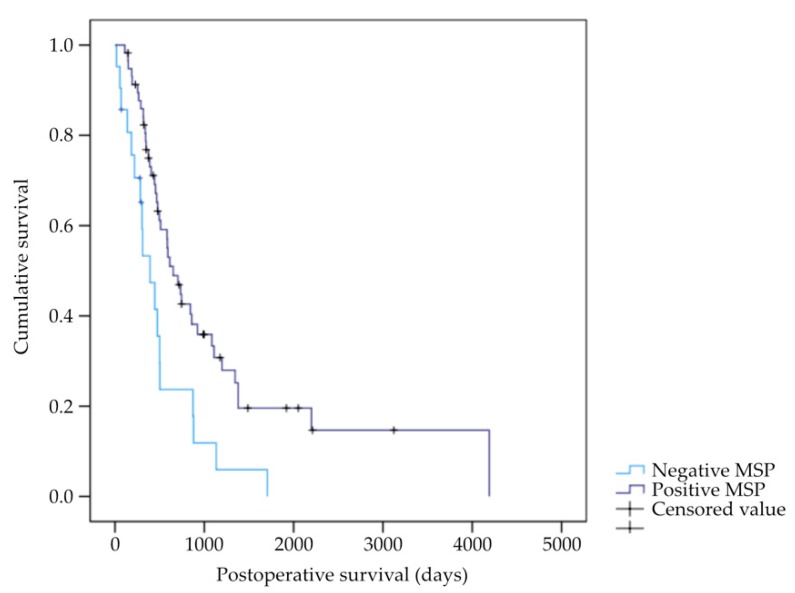
Kaplan–Meier plot for pancreatic cancer postoperative survival stratified by methylation-specific PCR (MSP) results concerning the status of ZNF154 methylation (*n* = 80, *p* = 0.004).

Specimens were subjected to bisulfite conversion and then analyzed using methylation-specific PCR. PANC-1 DNA was used as a positive control while DNA from HPDE-E6E7 cells served as a negative control. A total of 59 (73.75%) patients exhibited positive ZNF154 promoter methylation (MSP-positive) while only 21 (26.25%) patients did not have promoter methylation (MSP-negative). After analysis using the log rank test, we found that there was a significant correlation between the status of ZNF154 promoter hypermethylation and postoperative survival: MSP-positive patients had a markedly increased postoperative survival, with a median of 652 days, versus MSP-negative patients, who had a median postoperative survival of 392 days. Other clinical parameters, such as gender, age at the time of procedure, tumor size, histologic grading, status of lymph node, or distant metastasis, were not significantly correlated. Data are shown in Figure 2 and Table 2 and Table 3.

### 2.4. In Vitro Viability Assays

**Figure 3:**
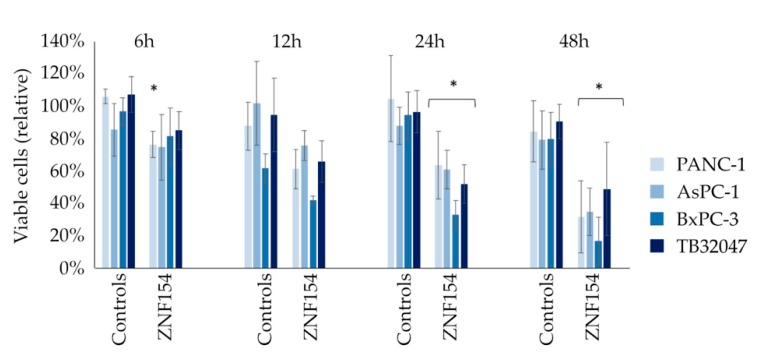
Percentages of viable cells following transient transfections with ZNF154 in PANC-1, AsPC-1, BxPC-3, and TB32047 cells after 6, 12, 24, and 48 h (* = *p* < 0.05 using student′s *t*-test).

The effect of ZNF154 hypermethylation on a cellular level was further characterized by in vitro experiments. Transient transfections with plasmids holding ZNF154-DNA were performed in four different cell lines. Automated cell counts of vital cells were conducted subsequent to transfections. PANC-1, AsPC-1, and BxPC-3 cells were used in the promoter hypermethylation analysis described above and are human pancreatic cancer cell models. While BxPC-3 is a wildtype, PANC-1 and AsPC-1 both carry a KRAS mutation. TB32047 is a murine pancreatic tumor cell line with a KRAS mutation. Transfections with the ZNF154 plasmid caused a significant reduction (*p* < 0.01) of vital cells in all cell lines after 24 and 48 h compared to the negative control (empty vector). The most prominent reduction of living cells was observed in BxPC-3 cells, as depicted in Figure 3. The efficiency of transient transfections was monitored by transfecting a plasmid (pcz-CFG5.1) coding for green fluorescent protein (GFP). The percentage of detected GFP in transfected cells ranged from 54% to 85%, which we judged as sufficient.

### 2.5. Quantification of RNA Transcript and Transcript Sequencing

**Figure 4:**
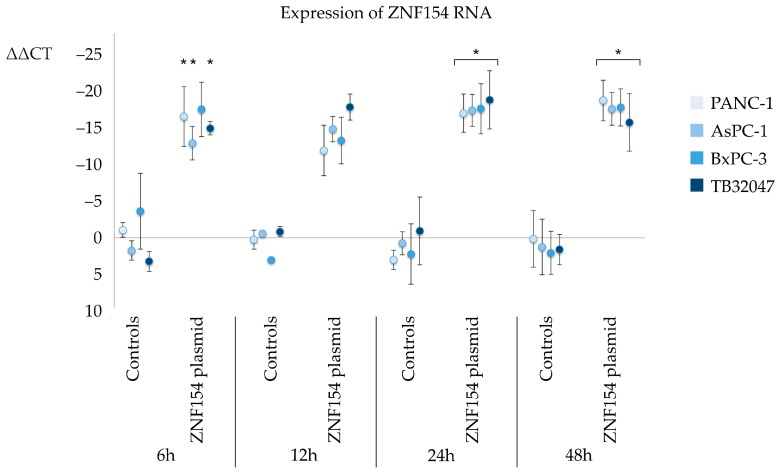
Relative expression of ZNF154 following transient transfections detected by *q*PCR (* = *p* < 0.05 using student′s *t*-test; ΔΔCt in relation to empty controls; error bars indicate standard deviation).

Transfected cells were analyzed with quantitative real-time PCR (*q*PCR) to check for levels of RNA expression. There was significant increase in the expression of ZNF154-RNA in all cell lines, which was most prominent after 24 and 48 h. The ΔΔCT values were −17 ± 3 at 24 h and −20 ± 3 at 48 h in PANC-1 cells, −18 ± 2 at 24 and 48 h in AsPC-1 cells, −18 ± 4 at 24 h and −19 ± 3 at 48 h in BxPC-3 cells, and −20 ± 4 at 24 h and −17 ± 4 at 48 h in TB32047 cells (Figure 4).

Sequencing was done with PANC-1 cells and AsPC-1 cells that had been transfected with either the ZNF154-plasmid or the pcz-CFG5.1-plasmid. Further gene analysis was performed on genes that exhibited a significant log2 fold change ≥ +2 or ≤ −2 following transfection. As a result, we identified three genes for further analysis: *SLFN5*, *TNFSF10*, and *LTB*. *SLFN5* was identified to have increased expression (PANC-1 + 2.5, AsPC-1 + 2.6). *ZNF154* revealed a strong induction in accordance with the prior results of *q*PCR. Data are displayed in Table 4. For confirmatory RNA quantitation, we conducted *q*PCR following transient transfection with ZNF154 in the aforementioned cell lines. It was revealed that expression of *LTB* was increased, not decreased, in PANC-1 cells after 48 h. Results for the expression of *LTB* in other cell lines or of *TNFSF10* in any cell line were not significant. There was a significant induction of *SLFN5* in PANC-1 and AsPC-1 cells after 24 h and also in BxPC-3 cells after 48 h.

### 2.6. Protein Expression

**Figure 5:**
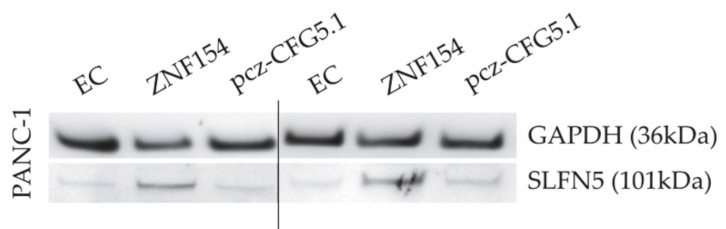
Western blots of PANC-1 cells transiently transfected with empty control (EC), ZNF154, or pcz-CFG5.1 (positive control).

We further investigated if transient transfections with the ZNF154 plasmid would affect protein levels of SLFN5. Transfections with empty controls, the ZNF154 plasmid, and the pcz-CFG5.1 plasmid as positive controls were carried out in PANC-1 cells. The SLFN5 protein runs were at 101 kDa. In PANC-1 cells, the SLFN5 band emitted a more intense signal as compared to controls after 24 and 48 h (Figure 5). 

## 3. Discussion

Pancreatic cancer resembles an aggressive tumor with poor prognosis. The short survival is mostly due to the lack of effective therapeutic means and late diagnosis. In earlier publications, we suggested an examination of pancreatic tumor cells for aberrant DNA methylation in order to identify novel molecular markers [8,9]. Sánchez-Vega et al. [10] confirmed methylation of the gene *ZNF154* in a variety of solid tumors. *ZNF154* encodes for a zinc-finger protein. Since zinc-finger proteins are, among other functions, involved in transcriptional activation and regulation of apoptosis [12], *ZNF154* has piqued our interest.

In the current study, we demonstrated that the ZNF154 promoter was hypermethylated in many pancreatic cell lines. This finding led to the assumption that ZNF154 hypermethylation might also be present in pancreatic cancer patients.

To investigate a possible presence of ZNF154 hypermethylation in vivo, we analyzed pancreatic cancer specimens from 80 patients. The patient collective reflects a typical surgical patient group. Almost three quarters (73.75%) of the patient collective had ZNF154 promoter hypermethylation. When we analyzed the patient data for the clinical course, we found a significant association between ZNF154 promoter hypermethylation and better postoperative survival (Figure 3, Table 2 and Table 3). A recent study has shown that ZNF154 methylation was associated with better survival in nasopharyngeal carcinoma [13]. In contrast, a different research group reported poor outcomes in loss of function of ZNF154 in prostate cancer [14]. Generally, ZNF154 promoter hypermethylation has been found in several cancer entities and was suggested for screening [10,15,16,17,18]. Pancreatic cancer is usually diagnosed at a late stage. This is due to the occurrence of little symptoms during the initial progress of the disease [19,20]. Hence, the usefulness of ZNF154 in screening for pancreatic cancer should be reassessed with a different patient collective for this matter. Few publications, however, have been able to implicate the prognostic value of ZNF154. In the present study, we demonstrated a positive correlation between silenced ZNF154 and prolonged survival in resectable pancreatic cancer patients. Future research in different tumor entities should target patient survival in relation to methylated ZNF154 as well.

The fact that ZNF154 promoter hypermethylation was the single factor associated with survival in our study underlines the need to investigate the basic molecular mechanisms of this disease. We evaluated the effects of ZNF154 in vitro with transient transfections in cells that were associated with a suppression of the ZNF154 promoter. We found that the artificial re-induction of ZNF154 by transient transfections in these cells led to increased cell death compared to transfected controls. While KRAS mutation status was independent of this finding, we demonstrated increased ZNF154 RNA levels in all cell lines. Generally, cell death or apoptosis is a mechanism of anti-cancer defense. However, cell death also increases pressure on clonal selection and ultimately contributes to higher sub-clonal variability. The current literature suggests that generated tumor evolution by cell death is associated with higher chemoresistance, more aggressive tumor progression, and a higher chance of tumor relapse [21]. Hence, silencing of ZNF154 might foster the growth of more stable, less aggressive tumor clones.

Complete sequencing of ZNF154 over-expressing cells disclosed three putative genes of interest. Further testing indicated a role for the gene *SLFN5*. Results for the remaining two genes were ambiguous or insignificant. Transfecting cells with ZNF154 induced significantly increased expression of *SLFN5* code as well as SLFN5 protein. Hence, we argue that *SLFN5* might play a role in pancreatic cancer and is connected to up- or downregulation of the *ZNF154* gene.

*SLFN5* is a member of the Schlafen family (*Slfn*) genes. These genes typically exhibit anti-neoplastic effects and are upregulated in response to interferon. The precise molecular mechanisms of *SLFN5* are unclear while its role in tumor growth has been fairly characterized. Few studies observed a positive correlation between *SLFN5* and shorter overall survival [22,23]. Yet, most authors report a connection between *SLFN5* and longer survival or less invasive growth, underlining that *SLFN5* is a tumor suppressor [24,25,26,27]. These findings concur with our observation that increased survival in pancreatic cancer patients is linked to silenced *ZNF154*, which, in turn, might translate into lower levels of *SLFN5*.

Taken together, we are the first, to our best knowledge, to report an association between hypermethylation of the ZNF154 promoter and better survival in resectable pancreatic cancer. Moreover, we suspect a link between a silenced *ZNF154* and *SLFN5* in pancreatic cancer. However, the exact mechanism between ZNF154 and SLFN5 remains to be elucidated.

## 4. Materials and Methods

### 4.1. Cell Culture

HPDE-E6E7 cells were a gracious gift from Dr. Ming Tsao, Toronto, Canada. TB32047 cells were a gracious gift from Dr. David Tuveson, CSHL, New York, NY, USA. PaCaDD cell lines were already available at the Research Laboratory of the Department for Visceral-, Thoracic-, and Vascular Surgery, University Hospital Carl Gustav Carus Dresden, Germany. All other cell lines were commercially acquired from ATCC, USA.

AsPC-1 cells and BxPC-3 cells were grown in RPMI 1640 medium supplemented with 10 mM Hepes, 1 mM sodium pyruvate, 4.5 g/L glucose, and 10% fetal bovine serum (FBS). TB32047 cells were grown in DMEM supplemented with 10% FBS. PANC-1 cells were grown in RPMI 1640 medium supplemented with 10% fetal bovine serum. All other cell lines were grown in either standard cell medium or in medium as recommended by the manufacturer. For transfer to further testing, cells were washed with DPBS, lysed with Trypsin-EDTA (0.25%) for 10 min, and suspended in FBS.

### 4.2. Patient Specimens

The study was conducted at the Department for Visceral-, Thoracic-, and Vascular Surgery, University Hospital Carl Gustav Carus Dresden, Germany. It was reviewed by the Ethics Commission (EK59032007) and was conducted ethically in accordance with the World Medical Association Declaration of Helsinki. All experiments adhered to the German Genetic Engineering Act (project number 55-8811.72/83/301 1 August 2002) as well as the data confidentiality principles of the Medical Faculty of the Technical University Dresden and the University Hospital Carl Gustav Carus Dresden. Informed consent to provide tissue for research and publication purposes was obtained from all participants prior to surgery.

To be included in the patient cohort, tissue samples had to be from patients diagnosed with ductal adenocarcinoma of the pancreas who received pancreatic resection at the University Hospital Carl Gustav Carus Dresden. Exclusion criteria were an insufficient DNA concentration of the sample (<2 ng/μL), incomplete bisulfate conversion, missing data, loss to follow-up, or postoperative survival of less than 14 days. Patient specimens were stored at the Biobank of the University Cancer Center of the University Hospital Carl Gustav Carus Dresden, Germany.

### 4.3. Methylation-Specific PCR (MSP) with Tumor Specimens and Cell Lines

Patient samples were either received as cryopreserved or paraffin-embedded tissue sections. These sections were probed using a 20-G needle. Cells from cell culture were collected after the lysis procedure described above and centrifuged. The resulting cell pellets were collected and washed with DPBS. Tissues or cell pellets were transferred into 1.5-mL Eppendorf tubes with 180 μL of ATL buffer and 20 μL of Proteinase K. Tubes were incubated on a thermo-shaker for 3 h at 56 °C and 700 rpm. In total, 4 μL of RNase A (100 mg/mL) was added and probes were incubated for 2 min at room temperature. Then, 200 μL of AL buffer was added and probes were incubated on a thermo-shaker for 10 min at 70 °C and 700 rpm. Next, 200 mL of ethanol (96–100%) was added and the lysates were transferred into QIAamp Mini columns (Qiagen, Hildesheim, Germany). Further DNA purification was carried out by following the instructions of the manufacturer. The final DNA suspension was transferred into 1.5-mL Eppendorf tubes, the DNA concentration was quantified by absorbance measurement, and tubes were stored at −20 °C.

Bisulfite treatment was accomplished using the EZ DNA Methylation-Gold Kit (Zymo, Freiburg, Germany) according to the manufacturer′s instructions.

For MSP, 20 ng of converted DNA aliquots were transferred in 0.5-mL Eppendorf tubes. Every MSP series included PANC-1 DNA as the positive control, HPDE6E7c7 DNA as the negative control as well as a master mix as the empty control. To exclude unsuccessful bisulfate conversion, we compared each MSP run with additional amplification results using HB14 primers [28]. Primer sequences and PCR cycler settings are available from the authors on request.

### 4.4. Quantitative Real-Time PCR (qPCR) of mRNA

Supernatant was taken from cell cultures, washed with PBS, and pipetted in 24-well plates. Further mRNA purification was accomplished using the miRNeasy Mini kit following the instructions of the manufacturer (Qiagen, Hildesheim, Germany). The obtained RNA samples were transcribed and amplified using the High Capacity cDNA Reverse Transcription Kit (ThermoFisher, Langenselbold, Germany) according to the manufacturer′s instructions. The final aliquots for *q*PCR were composed of 12.5 μL of Power SYBR Green Master Mix (ThermoFisher, Langenselbold, Germany), 2.5 μL of forward primer, 2.5 μL of reverse primer, 0.5 μL of DEPC-H_2_O, and 2 μL of cDNA. Housekeeping gene ACTB primers were used for normalization. *q*PCR was performed with the StepOne Real Time PCR System. Analysis was done with the Step One software (ThermoFisher, Langenselbold, Germany). Primer sequences are available from the authors on request.

### 4.5. Transient Transfections

Cell cultures were washed with DPBS and lysed with trypsin-EDTA. Approximately 3 × 10^4^ cells per well were plated with 500 μL of cell culture medium in 24-well plates and incubated for 24 h. The pcz-CFG5.1 plasmid was a gracious gift from Prof. Dr. Achim Temme, Dresden, Germany. All other plasmids were commercially acquired from Source Bioscience (Nottingham, UK) (clone accession BC160120 in poLEYFP-C1am vector) and were prepared with the Qiagen EndoFree Plasmid kit according to the manufacturer′s instructions (Qiagen, Hildesheim, Germany). Empty controls were solely treated with 100 μL of Opti-MEM medium (ThermoFisher, Langenselbold, Germany). ZNF154 plasmid aliquots were prepared with 91 μL of Opti-MEM, 1 μL of Lipofectamine 2000 (ThermoFisher, Langenselbold, Germany), and 8 μL (100 ng/μL) of ZNF154 DNA plasmid. Plasmid controls were prepared with 91 μL of Opti-MEM, 1 μL of Lipofectamine, and 8 μL (100 μg/μL) of pcz-CFG5.1 DNA plasmid. Following transfection, wells were incubated for 20 min at room temperature and analyzed after 6, 12, 24, 48, and 72 h for cell count, gene, and protein expression.

### 4.6. Cell Viability Assessment

Supernatant was removed. Cells were washed with 500 μL of DPBS, lysed in 150 μL of trypsin-EDTA, and resuspended in 500 μL of cell culture medium. Samples of 10 μL were transferred onto glass slides and analyzed with a TC20 Automated Cell Counter. Samples with cell counts below 5 × 10^4^ cells/mL were verified manually with a Neubauer slide.

### 4.7. RNA Sequencing

PANC-1 cells and AsPC-1 cells were analyzed after transient transfections with next-generation sequencing. mRNA was isolated from 1.5 ug of DNAse-treated total RNA using the Globin-Zero Gold rRNA Removal Kit (human, mouse, rat) from Illumina according to the manufacturer′s instructions. After elution in nuclease free water, a second poly-A targeted mRNA isolation using the NEBnext mRNA Magnetic Isolation Module (New England Biolabs GmbH; Frankfurt am Main Germany) was performed. Samples were then directly subjected to the workflow for strand specific RNA-Seq library preparation (Ultra Directional RNA Library Prep, NEB). For ligation, custom adaptors were used 1: (Adaptor-Oligo 5′-ACA CTC TTT CCC TAC ACG ACG CTC TTC CGA TCT-3′, Adaptor-Oligo 2: 5′-*p*-GAT CGG AAG AGC ACA CGT CTG AAC TCC AGT CAC-3′). In the following PCR enrichment (15 cycles), custom amplification primers were used carrying the index sequence indicated with ′NNNNNN′. (Primer1: Oligo_Seq AAT GAT ACG GCG ACC ACC GAG ATC TAC ACT CTT TCC CTA CAC GAC GCT CTT CCG ATC T, primer2: GTG ACT GGA GTT CAG ACG TGT GCT CTT CCG ATC T, primer3: CAA GCA GAA GAC GGC ATA CGA GAT NNNNNN GTG ACT GGA GTT. After XP bead purification, libraries were quantified using the Qubit dsDNA HS Assay Kit (ThermoFisher, Langenselbold, Germany), equimolarly pooled, and subjected to 75-bp single read sequencing on Illumina HiSeq 2500. RNA sequences were compared to the GRCh37 human reference genome using the Genomic Short-read Nucleotide Alignment Program [29]. RNA reads were counted with featureCounts [30] and analyzed for differential expression with DeSeq2 [31]. Significant findings were studied with PANTHER and WebGestalt [32,33].

### 4.8. Western Blots

Supernatant was removed. Cells were washed with 500 μL of DPBS, lysed in 150 μL of trypsin, incubated for 10 min at 37 °C, and resuspended in their specific cell culture medium. Aliquots of 1.5 mL each were prepared with collected cells in Eppendorf tubes and centrifuged at 3000× *g* for 5 min at room temperature. Supernatant was removed, cell pellets were washed with 500 μL of DPBS, and centrifuged at the same settings again. The cell pellets were buffered in 30 to 50 μL of RIPA buffer (containing protease inhibitor 1:100, phosphate inhibitor 1:100), put on ice for 30 min, treated in an ultrasound bath for 10 min, and centrifuged at 11,000× *g* at 4 °C for 20 min. The supernatant was collected in 1.5-mL Eppendorf tubes. Colorimetric determination of the protein concentration was achieved with a BCA Protein Assay (ThermoFisher, Langenselbold, German) according to the manufacturer′s instructions. In total, 10 μg of protein solved in RIPA buffer was mixed with 5 μL of 4 × NuPage LDS sample buffer, 2 μL of 10 × NuPage LDS reducing agent, and 1 × NuPage LDS sample buffer to a total volume of 25 μL each. Samples were incubated for 10 min at 90 °C in a thermo mixer and loaded onto 4–12% Bis-Tris polyacrylamide gels in XCell SureLock Mini-Cell chambers (ThermoFisher, Langenselbold, German). Mini cells were filled with NuPage antioxidant, Precision Plus Protein All Blue-Standards, and running buffer according to the manufacturer′s instructions. Samples were separated at 180 V for 55 min. Gels were electroblotted onto nitrocellulose membranes at 30 V for 60 min in XCell SureLock Mini-Cell systems. Blots were stained with Ponceau S and washed with TBST. After blocking in TBST wash (2.5 g of instant milk, 50 mL of TBST) overnight at 4 °C, the membranes were probed with primary antibodies. GAPDH antibodies (Cell Signaling Technology Cat# 2118, RRID: AB_561053; 1:1000 in 5% BSA) were applied at room temperature for 1 h. SLFN5 antibodies (*Santa Cruz Biotechnology; sc-240891*; 1:1000 in 5% BSA) were applied at 4 °C overnight. Blots were washed with TBST and incubated with corresponding secondary antibodies ((R and D Systems Cat# HAF109, RRID: AB_357236 for SLFN5 and Cell Signaling Technology Cat# 7074, RRID: AB_2099233 for GAPDH; 1:1000 in 5% non-fat milk) for 1 h at room temperature. Blots were washed again with TBST and incubated with horseradish peroxidase and luminol (1:1, approximately 0.1 mL/cm^2^) reactant for 5 min. Signal detection was performed with a G: BOX XT4 (Syngene, Cambridge, UK).

### 4.9. Statistical Analysis

Statistical analysis was performed with SPSS Statistics by IBM Corporation, USA. We used unpaired two-sample student′s *t*-test and log rank tests. *p*-values < 0.05 were considered statistically significant.

## Figures and Tables

**Table 1:** Patient characteristics of the patient collective.

Patient Characteristics	Absolute		Relative
Gender (male/female)	42/38		52.5%/47.5%
TNM classification			
pT1	1		1.3%
pT2	10		12.5%
pT3	67		83.8%
pT4	2		2.5%
pN0	26		32.5%
pN1	54		67.5%
pM0	73		91.3%
pM1	4		5%
pMx	3		3.8%
AJCC stages (7th edition)			
Ia	-		-
Ib	5		6.3%
IIa	20		25%
IIb	47		58.8%
III	1		1.3%
IV	4		5%
unknown	3		3.8%
Histologic grades			
G1	3		3.7%
G2	42		52.5%
G3	33		41.3%
G4	2		2.5%
	Mean	Minimum	Maximum
Patient age at time of surgery (years)	65.7	40	80
Postoperative survival (days)	707.2	17	4190

**Table 2:** Logrank test analysis results for median postoperative survival (days) according to methylation-specific PCR (MSP) status of ZNF154 (*n* = 80, SE = standard error).

MSP	Days	SE	95% Confidence Level	
			Lower Limit	Upper Limit
Negative	392	94	209	575
Positive	652	101	454	850
Total	583	67	452	714

**Table 3:** Clinical parameters and their respective strength of association for survival in our patient collective (MSP = methylation-specific PCR).

Parameters	Definition	*p*-Value	Odds Ratio	95% Confidence Level	
				Lower Limit	Upper Limit
Gender	0 = female 1 = male	0.098	0.617	0.348	1.093
Patient age at the time of surgery	metric	0.268	0.982	0.952	1.014
Tumor size	pT1/2 = 1 pT3/4 = 2	0.308	0.613	0.239	1.571
Lymph node status	pN0 = 0 pN1 = 1	0.230	1.472	0.783	2.769
Metastases	pM0 = 0 pM1 = 1	0.208	0.498	0.168	1.475
Histologic grade	G1/2 = 1 G3/4 = 2	0.115	1.588	0.894	2.822
MSP status	negative = 0 positive = 1	0.002	0.392	0.215	0.715

**Table 4:** Sequencing results in either PANC-1 or AsPC-1 cells for the genes of interest *SLFN5*, *TNFSF10*, *LTB*, and *ZNF154*.

Gene	PANC-1 log2 Fold Change	PANC-1 *p*-Value	AsPC-1 log2 Fold Change	AsPC-1 *p*-Value
*ZNF154*	9.338	0.0001	13.187	0.0032
*SLFN5*	2.453	0.0001	2.259	0.0002
*TNFSF10*	−3.932	0.0009	−1.513	0.0345
*LTB*	−1.288	0.0139	−5.342	0.0355

## Abbreviations

PDAC	Pancreatic ductal adenocarcinoma
MSP	methylation-specific PCR
PCR	polymerase chain reaction
AJCC	American Joint Committee on Cancer
GFP	green fluorescent protein
*q*PCR	real-time PCR
